# Haematological parameters, natural regulatory CD4 + CD25 + FOXP3+ T cells and γδ T cells among two sympatric ethnic groups having different susceptibility to malaria in Burkina Faso

**DOI:** 10.1186/1756-0500-5-76

**Published:** 2012-01-27

**Authors:** Guillaume S Sanou, Régis W Tiendrebeogo, André L Ouédraogo, Amidou Diarra, Alphonse Ouédraogo, Jean-Baptiste Yaro, Espérance Ouédraogo, Federica Verra, Charlotte Behr, Marita Troye-Blomberg, David Modiano, Amagana Dolo, Maria G Torcia, Yves Traoré, Sodiomon B Sirima, Issa Nébié

**Affiliations:** 1 Centre National de Recherche et de Formation sur le Paludisme, Ouagadougou, Burkina Faso; 2 Polytechnic University of Bobo Dioulasso, Bobo Dioulasso, Burkina Faso; 3 Department of Immunology and Infection, Faculty of Infectious and Tropical Diseases, London School of Hygiene and Tropical Medicine, London, UK; 4 Unité CNRS 5164IFR66- Pathologies Infectieuses et Cancer, Bordeaux2, Bordeaux, France; 5 Department of Immunology, Wenner-Gren Institute, Stockholm University, Stockholm, Sweden; 6 Department of Public Health Sciences, University La Sapienza, Rome, Italy; 7 Malaria Research and Training Centre, University of Mali, Bamako, Mali; 8 Department of Clinical Physiopathology, Center of Excellence DENOTHE, University of Firenze, Florence, Italy; 9 University of Ouagadougou, Ouagadougou, Burkina Faso

## Abstract

**Background:**

Fulani ethnic group individuals are less susceptible than sympatric Mossi ethnic group, in term of malaria infection severity, and differ in antibody production against malaria antigens. The differences in susceptibility to malaria between Fulani and Mossi ethnic groups are thought to be regulated by different genetic backgrounds and offer the opportunity to compare haematological parameters, Tregs and γδT cell profiles in seasonal and stable malaria transmission settings in Burkina Faso. The study was conducted at two different time points i.e. during the high and low malaria transmission period.

**Results:**

Two cross-sectional surveys were undertaken in adults above 20 years belonging either to the Fulani or the Mossi ethnic groups 1) at the peak of the malaria transmission season and 2) during the middle of the low malaria transmission season. Full blood counts, proportions of Tregs and γδ T cells were measured at both time-points.

As previously shown the Fulani and Mossi ethnic groups showed a consistent difference in *P. falciparum *infection rates and parasite load. Differential white blood cell counts showed that the absolute lymphocyte counts were higher in the Mossi than in the Fulani ethnic group at both time points. While the proportion of CD4+CD25^high ^was higher in the Fulani ethnic group at the peak of malaria transmission season (*p *= 0.03), no clear pattern emerged for T regulatory cells expressing FoxP3^+ ^and CD127^low^. However CD3^+^γδ^+ ^subpopulations were found to be higher in the Fulani compared to the Mossi ethnic group, and this difference was statistically significant at both time-points (*p *= 0.004 at low transmission season and *p *= 0.04 at peak of transmission).

**Conclusion:**

Our findings on regulatory T cell phenotypes suggest an interesting role for immune regulatory mechanisms in response to malaria. The study also suggests that TCRγδ + cells might contribute to the protection against malaria in the Fulani ethnic group involving their reported parasite inhibitory activities.

## Background

The pathogenesis of malaria is complex and most likely entails immunologic and non immunologic mechanisms [1]. The malaria parasite undergoes both extracellular and intracellular stages in the host, and thus the human immune system has to mobilize both humoral and cellular arms of immune responses in the fight against this parasitic infection. Whereas humoral immunity is directed toward the extracellular stages which include sporozoites and merozoites, cell-mediated immunity (CMI), in which T cells play a major role, targets hepatic stages of the parasites and may also be involved in blood stage immunity [2,3]. Thus some peripheral blood cells (lymphocytes, neutrophils, basophils, natural killers, etc.) and lymphocyte subsets are thought to play a major role in malaria parasite control [4-7].

Considering clinical protection against malaria in resident populations of endemic areas, previous studies have shown consistent differences in *Plasmodium falciparum *infection rates, malaria morbidity, prevalence and levels of antibodies to various *P. falciparum *antigens between Mossi and Fulani ethnic groups, where the Fulani ethnic group individuals are clearly less parasitized, and less affected by the disease [2,3,8-10]. No differences in the use of malaria protective measures, known genetic factors of resistance to malaria, and sociocultural or environmental factors were demonstrated that could account for these findings [3,11]. The differences between the two groups were suggested to be due to a functional deficit in regulatory T cells, as reflected by lower expression of TGF_β_, TGFβRs, CTLA4, and FOXP3 genes in Fulani compared with Mossi ethnic group or European donors not exposed to malaria [12]. CD4^+ ^CD25^+ ^Tregs are induced rapidly in humans following *P. falciparum *infection and are associated with a burst of the transforming growth factor-β (TGF-β) production, decreased parasite-specific immune responses, and higher rates of parasite growth. IL-10 from CD4^+^CD25^-^Foxp3^-^CD127^- ^adaptive regulatory T cells modulates parasite clearance and pathology during malaria infection [13]. Similarly, gamma delta (γδ) T cells, phenotypically expressing the CD3 antigens and molecules and representing 1-10% of circulating lymphocytes, are thought to play a role in infectious diseases. Vδ2+ T cells, the major circulating T-cell receptor-γδ-positive (TCR-γδ+) T cell subset in healthy adults, are involved in immunity against many microbial pathogens [14]. It has been reported that humans with *P. falciparum *malaria exhibit a marked increase in the number of peripheral blood γδ T cells, which remain elevated for more than a month after treatment [15]. Subsequently it was reported that cloned γδ cells are cytotoxic for *P. falciparum in vitro *[16-18] and that γδ T cells inhibit *in vitro *growth of the asexual blood stages of *Plasmodium falciparum *by a granule exocytosis-dependent cytotoxic pathway that required granulysin [19]. γδT cells can carry out many diverse functions, but individual subsets within the population have more restricted effector properties, depending on their expressed TCRs [20]. γδ T cells have also been implicated in the pathogenesis of malaria [21]. Human gamma/delta T cells express cytolytic and pro-inflammatory molecules such as IFN-γ, TNF-α, TNF-β [22] suggesting an immunoregulatory role for these cells [23]. A similar functional specialization is observed in human γδT cells: human Vδ2+ T cells generally have high cytotoxic activity and produce high levels of IFNγ [24]. As blood pathogen, malaria parasites are prone to cause haematologic disorders affecting nutrients and cells pattern. In severe *P. falciparum *malaria haematologic disorders which are the most common complications, play a major role in these fatal complications. These changes involve red blood cells, leukocytes, and haemostasis [25]. Patients with patent parasitemia tended to have significantly lower white blood cell, red blood cell, platelet, and haemoglobin levels than those who were malaria-negative [26]. Haematological changes were mild in the first 24 hours, but continued to deteriorate for few days after anti-malarial therapy.

In this study we have assessed haematological parameters, Tregs and γδ T cell profiles in the Mossi and Fulani ethnic groups living in a seasonal and stable malaria transmission setting of Burkina Faso at two different time points during the malaria transmission period in order to evaluate seasonal variation of haematological parameters and lymphocytes subsets (Tregs and γδ T cells) distribution and their impact on malaria susceptibility.

## Methods

### Study area

Details descriptions of study area have been reported elsewhere [2,3]. The study was carried out in two rural villages near the town of Ziniaré (35 km northwest of Ouagadougou, the capital city of Burkina Faso) in a shrubby savanna of the Mossi plateau (≈ 300 m above sea level) belonging to the Sudan-Sahelian ecoclimatic zone (isohyets, 600-900 mm). Malaria transmission is intense during the rainy season from June-October and sporozoite inoculation rate is above one infective bite per person per night [27]. The malaria transmission is very low during the dry season from November to May when the sporozoite inoculation rate is near zero. Main malaria vectors are *Anopheles gambiae, An. arabiensis *and *An. funestus*. Two chromosomal forms of *An. gambiae *and *An. funestus *are sympatric in the study area [27]. The Mossi ethnic group is living in the village of Barkoundouba-Mossi which is at 5 km from the village of Barkoundouba-Peulh inhabited by the Fulani ethnic group. The Fulani ethnic group is a settled population, living in similar houses as Mossi ethnic group. However, they maintain their tradition of keeping cattle in their environment.

### Study population

Adults aged more than 20 years from the Mossi and Fulani ethnic groups were recruited under a protocol approved by the Ethical Committee for Biomedical Research of the Ministry of Health, Burkina Faso and by the Ethics Committee of World Health Organization (Geneva). The study was conducted in compliance with International Conference on Harmonisation's Good Clinical Practice principles, the Declaration of Helsinki and the regulatory requirements of Burkina Faso. Individual written informed consent was obtained from all participants. Individuals were eligible for inclusion in the study if they were found to be healthy at a general medical examination and gave written informed consent. Exclusion criteria included: i) Pregnant women confirmed positive by the home pregnancy test (HPT), ii) any confirmed or suspected condition of immunosuppressive diseases including HIV (no screening test was been done for this purpose) by the physician; iii) chronic administration (defined as more than 14 days) of immunosuppressant or other immune-modifying drugs at the beginning of the study (this included oral steroids and inhaled steroids, but not topical steroids); iv) any chronic diseases (cardio-vascular, hepatic and renal) suspected by the physician to cause any supplementary risk to the volunteer; v) any other circumstances and condition suspected by the physician to be a risk for the volunteer health.

### Study design and sample collection

This study was a cohort study that involved Fulani ethnic group (considered more resistant to malaria) and the Mossi ethnic group (considered more susceptible to malaria). Two cross-sectional surveys were carried out, one during the malaria high transmission season 2007 (October) and one during the low transmission season in 2008 (May) in both ethnic groups. A list of potential adult volunteers has been generated based on the general census database. After explaining the study procedures, only those adults willing to participate were invited to attend to the study scheduled visits. For each adult who fulfilled eligibility criteria, a medical history was recorded, axillary temperature measured, physical examination done. Blood samples were obtained for malaria thick/thin smears, 4 ml in EDTA tube for haematological full blood count and 12 ml in Heparinated tubes for immunological parameter measurements.

### Malaria diagnosis and haematological parameters analysis

Thick and thin blood films prepared from finger prick blood samples, were air-dried and stained with 5% Giemsa. Asexual and sexual parasites were counted separately and species differentiated. Malaria parasites were counted against 200 white blood cells (WBC). A slide was declared negative only after reading against 2000 WBC without observation of a malaria parasite. The parasite count was converted to a parasite density per μL of blood. A full blood count was performed using a semi-automated haematology analyzer (Pentra 60).

### Isolation of peripheral blood mononuclear cells (PBMC)

Peripheral blood mononuclear cells (PBMC) were isolated from heparinized venous whole blood by gradient centrifugation on Ficoll-Hypaque (Histopaque 1077: Sigma^® ^1077-1, St Louis, USA). The isolated PBMC were washed and adjusted to a concentration of 20 × 10^6 ^cells/ml in a freezing solution (20% DMSO and 80% FBS) and after 18-24 hours of temporary storage at-80°C freezer, the cells were then transferred to liquid nitrogen until analysis.

### Flow cytometry cells subset phenotyping and cytokines intracellular detection

Thawed PBMCs were stained for surface and intracellular markers. Fluorochrome-linked monoclonal antibodies were: anti-CD3 PE (Clone UCHT1, Beckman Coulter, Marseille France), anti-CD4 PC5 (Clone 13B8.2, Beckman Coulter, Marseille France), anti-CD25 FITC (Clone B1.49.9, Beckman Coulter, Marseille France), anti-CD127 PE (Clone, R&D Systems Minneapolis USA), anti-TCR Panγδ PC5 (Clone IMMU510, Beckman Coulter, Marseille France), anti-TCR Vδ2 FITC (Clone IMMU389, Beckman Coulter, Marseille France), anti-FoxP3 PE with fixation and Permeabilization kit (Clone PCH101, eBiocience), anti-IFNγ PE (Clone 25723, R&D Systems Minneapolis USA), anti-IL10 PE (Clone 25209, R&D Systems Minneapolis USA). Isotype-controls fluorochromes conjugated were used for positive and unspecific fluorescence threshold setting. PBMCs were stimulated for 3 h at 37°C, 5%CO_2 _for intracellular detection of cytokines. Mitogens as PMA (10 ng/ml) and Ionomycin (1 μM) and at the same time protein inhibitor Brefeldin A (10 μg/ml) was added. An amount of 1 × 10^6 ^PBMCs were use for staining procedure according to manufacturer's instructions and about 10^5 ^CD4+ were acquired. Acquisitions were done in a three-colour Flow cytometer Cyflow SL with a blue LASER (488 nm) (Partec GmbH, Münster Germany). Data acquisition and FCS files analysis were done on FloMax Software. Table 1 present the staining panel used for the phenotyping. We looked for FoxP3^+ ^Tregs as CD4, CD25 and intranuclear FoxP3 phenotype T cells subset based on the notion that CD4^+^CD25^+^Foxp3^+ ^subsets exhibit suppressive functions *in vitro *[28].

**Table 1:** Phenotyping panel used for cells subsets detection

Phenotyping Panel	Antibodies - fluorochromes
Ex-vivo staining	

T cells	CD3-PE/CD4-PC5/CD25-FITC

γδ T cells	CD3-PE/Panγδ-PC5/Vδ2-FITC

CD127 T cells	CD127-PE/CD4-PC5/CD25-FITC

FoxP3 T cells	FoxP3-PE/CD4-PC5/CD25-FITC

Stimulated cells	

IL10 secreting T cells	IL10-PE/CD4-PC5/CD25-FITC

IFNγ secreting γδ T cells	IFNγ-PE/Panγδ-PC5/Vδ2-FITC

### Data analysis

All data were reported in Microsoft Office Excel 2003 files. Statistical analysis was performed in Stata 9 Software.

Differences in parasitemia, cytokine levels, and bioluminescence between both ethnic groups and seasons were analyzed using the Student's *t*-test where indicated. For all statistical tests, *p *< 0.05 was considered significant.

## Results

### Description of study participants

During the first cross-sectional survey conducted at the peak of the malaria high transmission period 133 adults above 20 years were enrolled (73 Fulani and 60 Mossi). At the second cross-sectional survey, 86 volunteers were re-bled (44 Fulani and 42 Mossi). The sex ratio and the mean ages were similar between both ethnic groups during the surveys (Table 2). *P. falciparum *indexes were higher in the Mossi ethnic groups during both transmission periods and the difference was statistically significant (*p *= 0.02) during the low transmission and *p *< 0.01 during the peak of malaria transmission. *P.falciparum *positive density in Mossi ethnic group were 733 (417-1050) and in Fulani ethnic group 405 (0-947).

**Table 2:** Description of study participants and full blood count in both ethnic groups at two time points of malaria transmission season

	Low Transmission Season			High Transmission Season		
	
	Fulani ethnic group (n = 44)	Mossi ethnic group (n = 42)	p** value	Fulani ethnic group (n = 73)	Mossi ethnic group (n = 60)	p** value
	
	% (95% CI)*	% (95% CI)*		% (95% CI)*	% (95% CI)*	
Male/Female	0.85	0.94	0.76	0.78	0.94	0.61

Mean age yrs*	33.9 (32.1-35.6)	34.9 (32.1-37.7)	0.56	33.0 (31.3-34.8)	33.3 (30.7-36.1)	0.52

Prevalence*	0,02	12.2 (1.7-22.7)	0.02	8.8 (1.9-15.7)	27.1 (15.4-38.0)	**< 0.01**

WBC (×10^3^)	5.2 (4.6-5.7)	5.7 (5.3-6.1)	0.10	5.3 (4.9-5.6)	6.1 (5.7-6.5)	**0.001**

RBC (×10^6^)	4.7 (4.5-4.8)	4.7 (4.6-4.9)	0.65	4.5 (4.3-4.6)	4.6 (4.4-4.7)	0.30

Haemoglobin	12.8 (12.1-13.5)	13.4 (12.8-13.9)	0.22	12,1 (11.5-12.7)	13.0 (12.6-13.5)	0.01

Platelets (10^3^)	217 (192.6-241.4)	209 (188-232)	0.66	182 (164-200)	199 (178-221)	0.21

Neutrophils (10^3^)	2.2 (1,8-2.5)	2.0 (1.8-2.2)	0.40	2.3 (2.1-2.6)	2.6 (2.3-2.8)	0.15

Lymphocytes (10^3^)	2.3 (2.0-2.5)	2.7 (2.5-3.0)	**< 0.01**	2.2 (2.0-2.4)	2.6 (2.4-2.8)	**0.01**

Monocytes (10^3^)	0.4 (0.3-0.4)	0.4 (0.4-0.5)	0.40	0.4 (0.3-0.4)	0.6 (0.5-0.6)	**< 0.001**

Basophils (10^3^)	0.04 (0.03-0.05)	0.04 (0.04-0.05)	0.31	0.04 (0.03-0.04)	0.04 (0.04-0.05)	0.30

Eosinophils (10^3^)	0.3 (0.3-0.4)	0.5 (0.3-0.7)	**0.03**	0.3 (0.2-0.3)	0.3 (0.3-0.4)	0.30

*95% CI, **p values calculated using the Student's *t*-test

Except for haemoglobin levels, which were higher in Mossi ethnic group during the peak of malaria transmission (*p *= 0.01), red blood cells counts were similar in both ethnic group during the two time points studied. White blood counts were higher in the Mossi than the Fulani ethnic group and this difference was statistically significant during the malaria high transmission season (*p *= 0.001) but not during the malaria low transmission season (*p *= 0.10). Differential white blood cell counts showed that the absolute lymphocytes counts were higher in Mossi than in Fulani ethnic group during both transmission periods (Table 3). Neutrophils and basophils did not differ between ethnic groups, or during the transmission seasons. Absolute monocyte counts were higher in the Mossi ethnic group at the peak of malaria high transmission (*p *< 0.0001), while it was similar in both ethnic groups during the low transmission season. Eosinophils count was higher during the low transmission season in the Mossi ethnic group but did not differ during the high malaria transmission season.

**Table 3:** Percentages of Tregs subpopulations among Fulani and Mossi ethnic groups at two time points of malaria transmission season

	Low transmission season			High transmission season		
	
	Fulani ethnic group (13)	Mossi ethnic group (23)	P** value	Fulani (ethnic group 24)	Mossi ethnic group (35)	P** value
	
	% (95% CI)*	% (95% CI)*		% (95% CI)*	% (95% CI)*	
%CD4^+^CD25^+ ^of CD3	4.12 (2.67-5.56)	4.11 (3.59-4.63)	0.99	5.28 (3.98-6.57)	4.52 (4.02-5.01)	0.20

%CD4^+^CD25^+ ^of CD4	7.23 (4.71-9.75)	6.74 (5.93-7.55)	0.63	8.44 (6.77-10.11)	7.10 (6.21-7.99)	0.12

%CD4^+^CD25^high^	3.30 (1.65-4.94)	2.75 (1.99-3.51)	0.47	2.92 (1.99-3.85)	1.91 (1.47-2.35)	0.03

%CD127^low^CD25^+^	2.59 (0.53-4.64)	1.93 (0.99-2.86)	0.48	0.96 (0.28-1.64)	0.79 (0.59-0.98)	0.63

%CD4^+^CD25^+^Foxp3^+^	2.03 (1.23-2.83)	1.62 (0.92-2.33)	0.42	1.83 (1.23-2.43)	1.98 (1.56-2.40)	0.67

%Foxp3^+^CD25^+ ^of CD4^+^CD25^+^	29.97 (20.34-39.59)	33.88 (28.51-39.26)	0.42	40.67 (35.87-45.48)	36.86 (31.84-41.88)	0.26

%CD4^+^IL10^+ ^of CD4	2.09 (0.06-4.25)	1.66 (1.00-2.32)	0.60	7.29 (5.16-9.41)	5.23 (3.89-6.57)	0.08

%CD25^+^CD4^+^IL10^+ ^of CD4^+^IL10^+^	54.91 (49.47-60.35)	51.64 (42.44-60.84)	0.6	31.32 (27.77-34.87)	23.69 (16.20-31.18)	0.07

%CD4^+^IL10^+ ^of CD25+	19.35 (8.98-29.72)	19.18 (9.74-28.62)	0.98	53.24 (43.85-62.63)	35.77 (27.33-44.20)	**0.006**

*95% CI, **p values calculated using the Student's *t*-test

### Regulatory T cells (Tregs) phenotypes

**Figure 1:**
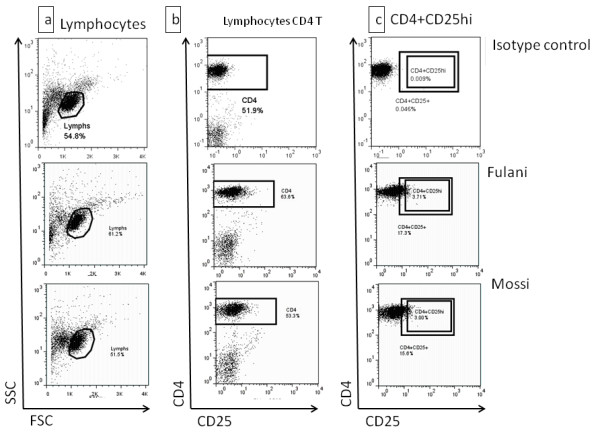
**Surface staining for CD3+CD4+CD25high T cells**. Ex-vivo thawed PBMCs were stained for immunophenotyping of T cells Subsets with a crucial role in immune response modulation. **a**) Lymphocytes were gated based on their light scatter characteristics in both ethnic groups. **b**) and **c**) Unstained cells and isotype control are used for positive events threshold determination in CD25 channel. Then based on fluorescence intensity level, CD25 high subsets were drawn.

**Figure 2:**
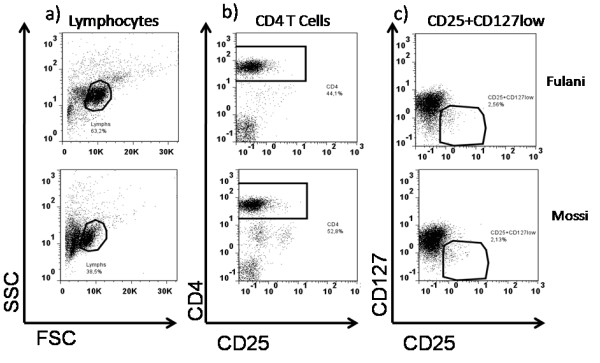
**CD4+ CD25+CD127low surface staining**. CD4+CD25+ regulatory T cells are known to downregulate CD127 antigen cells surface. This is correlate with FoxP3 production. Present gating strategy allows us to determine proportion of CD25+CD127 Low/- in our study subject's samples. **a**) Lymphocytes determination lead us to CD4 T lymphocytes (**b**) in white cells we to CD25+CD127low subsets cells (**c**).

**Figure 3:**
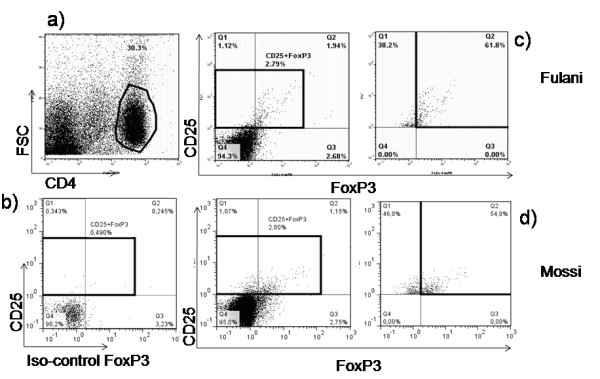
**FoxP3 Regulatory T cells immunophenotyping**. These cells express intranuclear FoxP3 protein that accessible by intracellular staining. A CD4 T Lymphocytes (**a**) gate display CD25+FoxP3+ T cells subset (**b**), (**c**) and (**d**) in Fulani and Mossi to access eventual difference these cells proportion between the two groups.

The frequencies of CD4^+ ^Tregs subsets among Fulani and Mossi ethnic groups at low and high transmission seasons are summarized in Table 3. A major difference between Fulani and Mossi was the frequency of CD4^+^CD25^+high ^cells that were predominant in Fulani group at the peak of malaria transmission season (2.92% in Fulani and 1.91% in Mossi) with a *p *= 0.03 (Figure 1), but remained similar at the low transmission season (3.3% in Fulani and 2.75% in Mossi, *p *= 0.47). Similar findings were observed for absolute numbers (Data not presented). Then we performed a second panel with also surface markers CD4, CD25 and CD127 assuming that CD25^+^CD127^low ^subset are proportional to Tregs population (Figure 2). CD4^+^CD25^+^CD127^low ^cells proportions between Fulani and Mossi ethnic group were not statistically different at both time point (Table 3) with 0.96% in Fulani and 0.79% in Mossi at high peak season (*p *= 0.63). In term of proportion of CD4^+^CD25^+^FoxP3^+ ^T cells, no differences between Fulani and Mossi ethnic group at low transmission (2.03% in Fulani and 1.62% in Mossi, *p *= 0,42) and high transmission (1.83% in Fulani and 1.98% in Mossi, *p *= 0.67) seasons were found (Figure 3).

**Figure 4:**
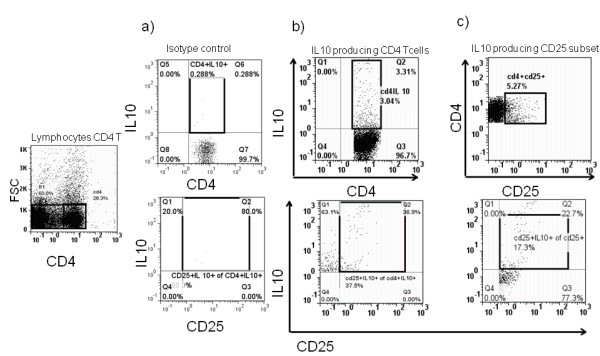
**Interleukin-10 (IL-10) secreting T cells Determination**. IL-10 is a cytokine with anti-inflammatory properties, has a central role in infection by limiting the immune response to pathogens. CD4+CD25+ producing Interleukin-10 were determined by gating on CD4 T cells on Light scatter and using isotype control (**a**). CD25 secreting IL10 were identified in all CD4 producing IL10 (**b**) and also in CD4+CD25+ T cells (**c**) to access proportion in all CD4 producing Interleukine -10 and proportion of those produce the cytokine among all CD25 subset.

Again we performed an *in vitro *stimulation for 3 hours with mitogens PMA/Ionomycin and protein translocation inhibitor Brefeldin A. We differentiated the percentage of CD4^+ ^producing IL10 as well as the CD4^+^CD25^+ ^phenotypes T cells secreting IL10. Although higher proportion of IL10^+^CD4^+^CD25^+ ^were observed in Fulani (7.29%) compared to Mossi (5.23%) ethnic group mainly at the high transmission season, the difference was not statistically significant (*p *= 0.07) (Figure 4).

### γδ T sub-populations

**Figure 5:**
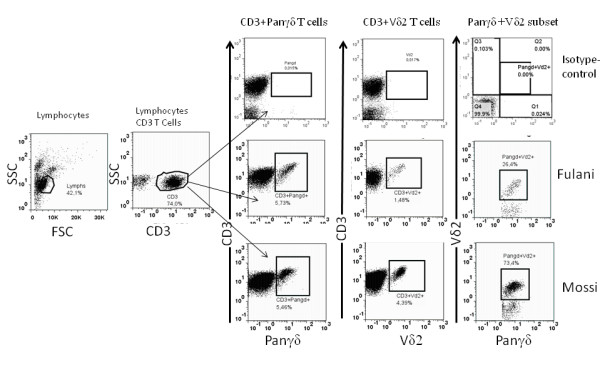
**Characterisation of γδ T cells**. a) Lymphocyte CD3 T cells were gated on total lymphocytes (a) based on light scatter characteristics. Then CD3 T cells proportion of Panγδ and Vδ2 subset (b) were access and Panγδ+Vδ2+ proportion of Panγδ T cells were also determined (c).

**Figure 6:**
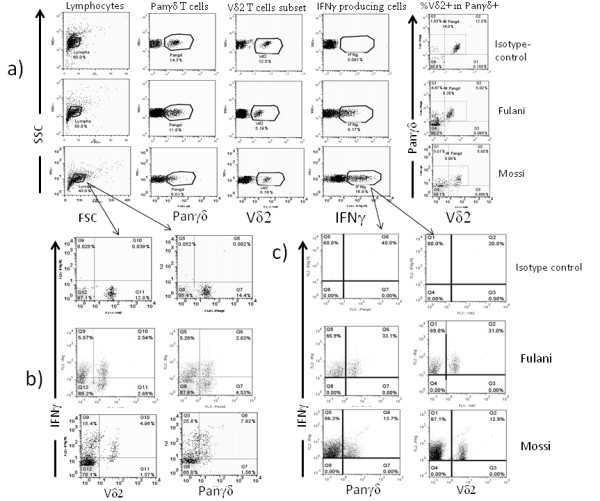
**Interferon-γ production in γδ T cells**. The present data show IFNγ secretion in γδ T cells after a short stimulation by mitogens (PMA/Ionomycin). We first access to the different subsets phenotypically (a) then their ability to express the cytokine (b) and the proportion among all Interferon-γ secreting cells.

In order to evaluate the eventual imbalance between Tregs and γδ T cells also known to be active on *Plasmodium *clearance, we performed a CD3, Panγδ(TCR) and Vδ2 panel. Data on γδ T cells subsets are summarized in Table 4. The proportions of CD3^+^Panγδ^+ ^(TCRγδ^+^) in Fulani ethnic group were higher compare to Mossi ethnic group (with respectively 13.23% and 5.59%). In Mossi ethnic group we noted that the CD3^+^Panγδ^+ ^proportion remains stable through the season, whereas in Fulani ethnic group the proportion of these cells was double of that measured in Mossi ethnic group at the low transmission season and this difference was highly significant (*p *= 0.004) (Figure 5) and remained significant during the high transmission season (*p *= 0.04). Although the percentages of Vδ2^+ ^predominant among circulating γδ T cells subsets were similar in Fulani compared to Mossi ethnic group during both surveys the differences did not reach statistical significance (*p *= 0.12 and p = 0.48 respectively). *In vitro *stimulation of PBMCs for IFNγ secretion by γδ T cells showed that Vδ2^+ ^IFNγ producing cells proportion (Figure 6) were similar in both populations at the two time point (Table 4).

**Table 4:** Percentages of γδ T cells phenotypes among Fulani and Mossi ethnic groups at two time points of malaria transmission season

	Low transmission season			High transmission season		
	
	Fulani ethnic group (12)	Mossi ethnic group (17)	P** value	Fulani ethnic group (37)	Mossi ethnic group (58)	P** value
	
	% (95% CI)*	% (95% CI)*		% (95% CI)*	% (95% CI)*	
%CD3 + Panγδ + of CD3+	13.23 (7.32-29.14)	5.59 (4.16-7.03)	**0.004**	8.07 (6.29-9.86)	5.86 (4.67-7.05)	0.**04**

%CD3 + Vδ2+ of CD3	4.30 (3.04-5.57)	3.09 (2.05-4.14)	0.1248	4.04 (3.03-5.06)	3.28 (2.43-4.14)	0.2487

%CD3+ Vδ2+ of CD3 + Panγδ+	25.55 (16.46-34.65)	35.39 (26.23-44.56)	0.1249	43.65 (37.13-50.17)	46.70 (40.87-52.53)	0.4823

%CD3+ Vδ2-of CD3 + Panγδ+	74.41 (65.34-83.49)	64.56 (55.40-73.73)	0.1244	56.28 (49.75-62.81)	53.80 (47.91-59.69)	0.5694

% Panγδ + of IFNγ+	17.45 (12.53-22.37)	15.71 (10.02-21.39)	0.6246	13.22 (1.03-25.41)	19.87 (11.05-28.69)	0.3952

%Vδ2+ of Panγδ + IFNγ+	60.25 (48.71-71.80)	55 (44.76-65.24)	0.4637	66.17 (46.06-86.29)	49.51 (38.25-60.77)	0.1151

%IFNγ of Vδ2+	38.3 (25.78-50.82)	35.33 (21.38-49.28)	0.7372	41.36 (23.88-58.85)	27.68 (18.67-36.70)	0.1112

%IFNγ + of Vδ2-	17.37 (11.72-23.03)	23.06 (12.87-33.26)	0.3227	12.89 (5.25-20.54)	22.10 (14.88-29.32)	0.1498

*95% CI, **p values calculated using the Student's t-tests

## Discussion

The aim of this study was to examine the haematological, Tregs and γδ profiles of two groups known to differ in their susceptibility to malaria to identify factors that might be associated with these differences. Mossi ethnic group exhibited higher *P. falciparum *prevalence and parasite load than the Fulani ethnic group during both high and low malaria transmission period. These results were consistent with those described in previous reports [2,3,9,10]. The capacity to generate stronger antibody responses against different epitopes of *P. falciparum *antigens has been reported to be a specific feature of Fulani ethnic group compared to Mossi and Rimaibé ethnic groups [2,3,9].

Due to our three-color cytometer, it was impossible to access directly CD3^+^CD4^+^CD25^+^FoxP3^+^CD127low subset phenotype which is the currently accepted definition of regulatory T cells [29,30]. We then performed a multiple tubes test.

Total WBC and total lymphocytes were higher in Mossi ethnic group, despite the high prevalence of malaria infection, When we compared Fulani and Mossi ethnic group volunteers according to their parasitemia status (positive or negative), no difference was seen may be due to relative low parasite density. There was no evidence for an association between infected and non infected volunteers (Data no shown). This is not consistent with previous findings where WBC and low lymphocytes counts were associated to malaria infection [26,31,32]. Although there is strong evidence showing the potential of malaria infection to affect the counts of lymphocyte subpopulations in the peripheral blood, this might not be consistent in all geographical locations. The pathogenesis as well as the disease outcome of malaria is highly dependent on local factors such as the level of endemicity, genetic background, nutritional status, demographic factors, malaria immunity, host genetics, and parasite factors as well [33-35]. Even if the higher lymphocyte counts cannot explain the susceptibility of Mossi ethnic groups to malaria, the differences observed in lymphocytes counts might be attributed to genetic diversities between the examined ethnic groups.

We also found that haemoglobin level, absolute monocyte counts were higher in the Mossi ethnic group with a statistical significance during the high malaria transmission season although the medical examination showed that all the volunteers were apparently healthy. This could be due to differences in the immune status of the study subjects related to the level of malaria endemicity, or it could be due to a possible difference in genetic background, physiological feature which may cause differences in cells activation [35].

Regulatory T cells are reported to be key mediators of peripheral immune homeostasis, and control tolerance to self antigens [36,37], and immune responses to environmental allergens, tumors and invading pathogens [38]. In recent years their contribution to immune evasion and immunopathology during malaria infection has been highlighted and they have been found to be increase during human malaria [13,28,39-42]. In this paper, the frequencies of the T cells CD4^+^CD25^+^FoxP3^+ ^subsets were similar in Fulani and Mossi ethnic groups at the low and high malaria transmission period. These data suggest that the generation of CD4^+^CD25^+^FOXP3^+ ^T cells is not compromised in Fulani ethnic group compared to Mossi ethnic group but they do not indicate whether these cells are endowed with full suppressive activity. A previous study in the same area by Torcia *et al*., reported that immune modulating genes (including TGFβ, TGFβRs, CTLA4 and FoxP3) were less expressed in CD4^+^CD25^+ ^cells of the Fulani ethnic group compared to Mossi ethnic group [12] as measured by microarray and RT-PCR techniques, suggesting that autocrine/paracrine circuits maintaining the regulatory phenotype, in particular those triggered by TGFβ are interrupted or less efficient in Fulani ethnic group. The authors also suggested that these defects are correlated with a defective function of CD25^+ ^cells in suppressing cell proliferation induced by malaria antigens.

An interesting data emerging from this phenotypical analysis is that the frequency of CD4+CD25+secreting IL10 is higher in the Fulani ethnic group compared to Mossi ethnic group. This IL10 producing subset of regulatory T cells with CD4+CD25+FOXP3 immune phenotype has been reported in murine models of malaria infection [13]. A possible interpretation is that alternative circuits of immune suppression were activated in Fulani groups due to the low or absent production of TGFβ by regulatory T cells. These findings are consistent with previous results by Torcia *et al*. in the same ethnic groups during the malaria high transmission period. In that study microarrays techniques were used, using RNA from CD4^+^CD25^+ ^cells, and they could show that immune modulating genes (including TGFβ, TGFβRs, CTLA4 and FoxP3) were less expressed in the Fulani ethnic group. The low activities of Tregs have also been emphasized by the lower rate serum level of TGFβ and higher concentration of proinflammatory chemokines CXCL10 and CCL2 in Fulani ethnic group. In addition depletion of CD25^+ ^Tregs the proliferative response of Fulani ethnic group to malaria specific antigens while this response was significantly increased in Mossi ethnic group [12]. The discrepancy observed between the two studies might be explained by the differences in the techniques used because the microarray technique is more sensitive and more specific than the phenotyping we used in our study. Moreover, the limited numbers of Tregs markers might be an handicap in the understanding of the role of these T cells subsets in our study populations because many studies suggest that FoxP3 may be a key regulatory factor of Tregs, controlling the expression of multiple genes that mediate an entire regulatory program [43,44].

Expansion of Vγ9/Vδ2^+ ^T cells is observed in many infectious diseases. The increase in γδ T Cells during the first decade of life is largely limited to the Vγ9/Vδ2^+ ^population, causing a shift towards the Vγ9 dominance seen in Caucasian adults [45]. The increased frequencies of γδ T Cells in African donors were not simply due to decreased numbers of Vγ9/Vδ2^+ ^cells in the African compared to the Caucasian donors but also due to Vδ2^- ^subset. In conclusion, the high proportion of TCR γδ Cells among peripheral CD3+ cells is mainly due to expansion of others Vδ^+ ^subsets. The phenotyping of γδ T cells subsets has revealed that the proportion TCR γδ+ population was significantly higher in Fulani ethnic group than in Mossi ethnic group. None of the subpopulations (Vδ2^+ ^or Vδ2^-^) showed a particular pattern in both ethnic groups. A similar trend was observed for the IFNγ+ secreting subsets. Although we were not able to establish a relationship between the parasite load and the frequency of TCRγδ^+ ^because of low parasite density and prevalence in our study population, the cytotoxicity role attributed to TCRγδ^+ ^against *P. falciparum *[16-19] might participate to the resistance of Fulani ethnic group to malaria. These findings suggest that different cells lines may affect the development of naturally acquired immunity against infectious diseases in these populations. The enhanced total lymphocyte counts and TCRγδ^+ ^cells support the hypothesis that the protection against malaria of Fulani ethnic group might be at least partially mediated by some T cells subset.

## Conclusion

Our findings on regulatory T cells phenotypes further on IL 10 producing cells suggest an interesting role of immune regulatory mechanisms in malaria response. The study also suggests that TCRγδ + cells might contribute to the protection against malaria in the Fulani ethnic group involving their reported parasite inhibitory activities.

## Competing interests

The authors declare that they have no competing interests.

## Authors' contributions

GSS participated in study design, samples collection, lab work, data analysis and manuscript preparation. TRW participated in study design, samples collection, lab analysis, data analysis and manuscript preparation. ALO participated in study design, lab analysis, data analysis and manuscript preparation. AD participated in lab analysis, data analysis and manuscript preparation. AO participated in study design, clinical collection and manuscript preparation. JBY participated in clinical collection and manuscript preparation. EO participated in clinical collection and manuscript preparation. FV participated in study design and manuscript preparation. CB participated in study design, data analysis and manuscript preparation. MTB participated in study design, data analysis and manuscript preparation. DM participated in study design, data analysis and manuscript preparation. Ado study design, data analysis and manuscript preparation. MGT participated in study design helped to draft the manuscript. YT participated in study design, data analysis and manuscript preparation. SBS participated in study design, data analysis and manuscript preparation. IN participated in study design, data analysis and manuscript preparation and overall study supervision. All authors read and approved the final manuscript.
